# Preclinical tissue distribution and metabolic correlations of vigabatrin, an antiepileptic drug associated with potential use‐limiting visual field defects

**DOI:** 10.1002/prp2.456

**Published:** 2019-01-07

**Authors:** Dana C. Walters, Erwin E. W. Jansen, Garrett R. Ainslie, Gajja S. Salomons, Madalyn N. Brown, Michelle A. Schmidt, Jean‐Baptiste Roullet, K. M. Gibson

**Affiliations:** ^1^ Department of Pharmacotherapy College of Pharmacy and Pharmaceutical Sciences Washington State University Spokane Washington; ^2^ Metabolic Laboratory Department of Clinical Chemistry Amsterdam University Medical Center Amsterdam The Netherlands

**Keywords:** β‐alanine, 4‐guanidinobutyrate, enantiomers, GABA, tissue distribution, Vigabatrin

## Abstract

Vigabatrin (VGB;* (S)‐(+)/(R)‐(‐)* 4‐aminohex‐5‐enoic acid), an antiepileptic irreversibly inactivating GABA transaminase (GABA‐T), manifests use‐limiting ocular toxicity. Hypothesizing that the active *S* enantiomer of VGB would preferentially accumulate in eye and visual cortex (VC) as one potential mechanism for ocular toxicity, we infused racemic VGB into mice via subcutaneous minipump at 35, 70, and 140 mg/kg/d (n = 6‐8 animals/dose) for 12 days. VGB enantiomers, total GABA and β‐alanine (BALA), 4‐guanidinobutyrate (4‐GBA), and creatine were quantified by mass spectrometry in eye, brain, liver, prefrontal cortex (PFC), and VC. Plasma VGB concentrations increased linearly by dose (3 ± 0.76 (35 mg/kg/d); 15.1 ± 1.4 (70 mg/kg/d); 34.6 ± 3.2 μmol/L (140 mg/kg/d); mean ± SEM) with an *S*/*R* ratio of 0.74 ± 0.02 (n = 14). Steady state *S*/*R* ratios (35, 70 mg/kg/d doses) were highest in eye (5.5 ± 0.2; *P* < 0.0001), followed by VC (3.9 ± 0.4), PFC (3.6 ± 0.3), liver (2.9 ± 0.1), and brain (1.5 ± 0.1; n = 13‐14 each). Total VGB content of eye exceeded that of brain, PFC and VC at all doses. High‐dose VGB diminished endogenous metabolite production, especially in PFC and VC. GABA significantly increased in all tissues (all doses) except brain; BALA increases were confined to liver and VC; and 4‐GBA was prominently increased in brain, PFC and VC (and eye at high dose). Linear correlations between enantiomers and GABA were observed in all tissues, but only in PFC/VC for BALA, 4‐GBA, and creatine. Preferential accumulation of the VGB *S* isomer in eye and VC may provide new insight into VGB ocular toxicity.

**Abbreviations**
4‐GBA: 4‐guanidinobutyric acid
BALA: alanine
CNS: central nervous system
GABA: γ aminobutyric acid
GABA‐T: GABA‐transaminase (also aminobutyrate aminotransferase)
GHB: γ hydroxybutyric acid
PFC: prefrontal cortex
SSADHD: succinic semialdehyde dehydrogenase deficiency
SSADH: succinic semialdehyde dehydrogenase
SSA: succinic semialdehyde
VC: visual cortex
VGB: vigabatrin

## 1 INTRODUCTION

**Figure 1:**
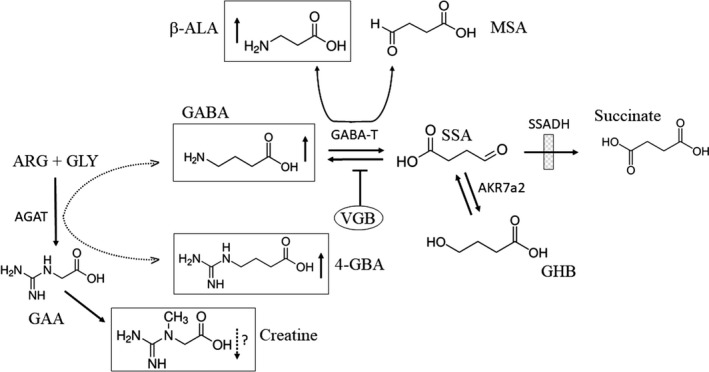
The metabolic pathway of GABA. GABA is converted to succinate in a two‐step sequence catalyzed by GABA‐transaminase (GABA‐T; generating succinic semialdehyde (SSA)) and succinic semialdehyde dehydrogenase (SSADH). The latter is the site of the defective enzyme in patients who manifest accumulation of γ‐hydroxybutyric acid (GHB) in body fluids (cross‐hatched box). Intermediates quantified in the current study included vigabatrin (VGB; irreversible inhibitor of GABA‐T), GABA, β‐ALA (β‐alanine), 4‐GBA (4‐guanidinobutyrate), and creatine. The latter is postulated to derive from interference of increased GABA in the AGAT (arginine amidinotransferase) reaction, which catalyzes the formation of guanidinoacetate (GAA) from ARG (arginine) and GLY (glycine). Additional abbreviations: AKR7a2, aldo‐keto reductase 7a2; MSA, malonic semialdehyde. Upward pointing arrows indicate predicted elevations in animal tissues with administration of VGB, and dashed lines indicate postulated reactions or predicted decreases, the latter in the case of creatine

The mechanism of action of vigabatrin (VGB), which directly impacts the GABA metabolic pathway by irreversible inhibition of GABA‐transaminase^1^ (Figure 1), is unique among antiepileptic drugs. VGB is the first‐line intervention in infantile spasms,^2^ yet it carries a black‐box warning indicating potential ocular toxicity, primarily associated with narrowing of the peripheral visual field and requiring routine visual testing during its use. VGB‐associated ocular toxicity has been broadly investigated without conclusive evidence for specific underpinning mechanisms. A detailed understanding of the off‐target effects of VGB in the eye, and pharmacotherapeutic approaches potentially mitigating this toxicity, would significantly extend the utility of this important drug.

Succinic semialdehyde dehydrogenase (SSADH) deficiency (SSADHD) is a rare heritable disorder of GABA metabolism (Figure 1) for which VGB intervention should be well‐suited. Ablation of SSADH activity, the second enzyme in the conversion of GABA to succinic acid (Figure 1), results in a neurometabolic disorder whose nonspecific neurological morbidity associates with accumulation of GABA and the GABA derivative γ‐hydroxybutyric acid (GHB), the latter a GABA analogue.^3^ Based upon the prediction that GHB in central nervous system (CNS) will decrease with VGB intake, it is often employed empirically in SSADHD. Indeed, metabolic studies in cerebrospinal fluid (CSF) derived from SSADHD patients before and after VGB intervention^4^ have documented decreased GHB and further enhanced GABA elevation. It is unknown if enhancement of GABA levels via VGB is prudent in patients already manifesting increased GABA, perhaps providing insight as to why clinical outcomes with VGB in SSADHD have been mixed.^5^ An additional concern with VGB intervention resides in its capacity to increase additional intermediates via GABA‐T inhibition, including homocarnosine (GABA:L‐histidine dipeptide),^6^ β‐alanine (a GABA‐T substrate; Figure 1),^7^ and 4‐guanidinobutyrate (4‐GBA),^8^ an intermediate purported to derive from GABA interference in the arginine glycine amidinotransferase reaction of creatine synthesis (Figure 1). Although GABA, and GABAergic mechanisms, have been implicated in VGB‐related ocular toxicity, the potential mechanistic role for other metabolites remains unclear.^9^, ^10^, ^11^

VGB formulations are racemic mixtures of the (*S)‐(+)* and (*R)‐(‐)* enantiomers (equal ratio), with only the (*S)‐(+)* enantiomer responsible for GABA‐transaminase inactivation and therapeutic activity^1^, ^12^. Consequently, it has been assumed that the reported ocular toxicity of VGB is likely related to the active (*S)‐(+)* enantiomer. The specific toxicity of VGB to the eye remains unexplained. Tissue sequestration of the (*S)‐(+)* enantiomer as a result of its irreversible binding to GABA‐T has been proposed as an explanation for its greater plasma exposure compared to the (*R)‐(‐)* enantiomer when administered at identical dose in healthy volunteers.^13^, ^14^ Hence, ocular toxicity could be the consequence of a greater residence time of the (*S)‐(+)* enantiomer in the eye than in other tissues, greater local activity and greater toxicity. Other studies have reported that only the (*S)‐(+)* enantiomer is actively transported into neurons and astrocytes, suggesting a greater uptake of the active enantiomer in the CNS including the anatomical and cellular components of the visual system. However, such a selective tissue uptake has not been demonstrated in vivo.^15^ One could also propose that in the eye, the (*R)‐(‐)* enantiomer is converted to the (*S)‐(+)* enantiomer, leading to a greater local inhibition of GABA‐T with further enhancement of toxicity. However, studies have shown that chiral inversion of VGB does not occur in vivo,^13^ Finally, one could speculate that the (*R)‐(‐)* enantiomer is responsible for the ocular toxicity of the drug. However, this enantiomer is believed to be inactive and its potential toxicity on the visual system has not been evaluated. To our knowledge, there are no reports of VGB enantiomer measurement in the eye or the visual cortex following racemic VGB administration

Here, we hypothesized that preferential and correlative accumulation of vigabatrin's active (*S)‐(+)* enantiomer and GABA‐derived metabolites in eye and/or visual cortex would occur, providing novel insight toward understanding the mechanism(s) of its selective visual field toxicity. To address this hypotheses, we performed tissue measurements of VGB enantiomers and GABA‐derived metabolites in mice (C57BL/6) infused with varying doses of racemic VGB. The results of these studies show preferential accumulation of the (*S)‐(+)*enantiomer in eye and visual cortex, underscoring potential novel metabolic and pharmacological underpinnings for VGB's selective visual field toxicity.

## 2 MATERIALS AND METHODS

### 2.1 Preparation and purification of VGB

Racemic VGB (catalog 0808/10) was obtained from Tocris Biosciences (Bristol, United Kingdom) and employed for assay of tissue VGB. For synthesis of larger quantities of racemic VGB, as required for in vivo studies, 5‐vinyl‐2‐pyrrolidone (29 g, 0.262 mol) was dissolved in a mixture of isopropanol (308 mL) and deionized water (30 mL) under Ar. To this solution, potassium hydroxide (22 g, 0.393 mol) was added at room temperature and the mixture was then heated at 75°C for 24 hours. After cooling, the reaction was quenched with glacial acetic acid (24 mL) and crude 4‐amino‐5‐hexenoic acid (VGB) precipitated in the reaction mixture. VGB was recovered by filtration and recrystallized in a mixture of water/isopropanol to give white 4‐amino‐5‐hexenoic acid (27.3 g, 81%, >98% purity by NMR). ^1^H NMR (MeOD, 400 MHz) δ (ppm) 5.92‐5.80 (m, 1H), 5.42 (d, J = 8.0 Hz, 1H), 5.39 (s, 1H), 3.75 (dd, J = 6.4, 13.6 Hz, 1H), 2.54‐2.25 (m, 2H), 2.05‐1.80 (m, 2H); LCMS (ESI) tR: min (>99%, ELSD), m/z: 130.3 [M + 1]+. Absolute verification of *S* and *R* VGB was achieved with *S* VGB standard (catalog V113) obtained from Sigma Aldrich (St. Louis, MO) using liquid chromatography‐tandem mass spectrometry (LC‐MS/MS).

### 2.2 Subcutaneous infusion of VGB to mice

Animal studies were carried out in accordance with the Guide for the Care and Use of Laboratory Animals as described by the NIH (OLAW; Office of Laboratory Animal Welfare). All procedures were approved by the Washington State University Institutional Animal Care and use Committee (Protocols ASAF 4232‐42 and 6134).

C57BL/6J mice (Jackson Laboratories, Bar Harbor, ME) were bred in‐house to obtain required cohort size (n = 6‐8, male only), and were 8‐10 weeks of age and 20.8‐26.1 g in weight. Osmotic minipumps, model 2002 (Alzet, Cupertino, CA) were prepared 18‐24 hours prior to surgery. The minipumps deliver drug at a constant flow rate of 0.5 μL/h for up to 14 days. To achieve the desired delivery concentrations of 35, 70, and 140 mg/kg/d VGB (based upon an average mouse weight of 25 grams), pumps were filled with vehicle (PBS) or 73, 146, and 291 mg/mL VGB solution in PBS respectively. These daily dosages of VGB were chosen to parallel both pediatric and adult clinical dosing. For pediatric dosing, VGB is begun at 25‐50 mg/kg/d, maintenance 50‐150 mg/kg/d, maximum 200 mg/kg/d. For adult dosing, intervention is started at 250 mg twice daily (~10 mg/kg/d), maintenance at 500‐1000 mg twice daily (total dose 1000‐2000 mg), and maximum at 1500 mg twice daily, equivalent to a 3000 mg total daily dose (~ 50 mg/kg/d).

Animals were randomly assigned to vehicle or drug cohorts. While filling the minipumps, care was taken to avoid air bubbles and fill the reservoirs completely (~200 μL). The loaded minipumps were placed in a sterile falcon tube containing 0.9% sterile saline until surgical implantation. Following preparation for surgery (alcohol/betadine scrub), a small incision was made in the neck region of each mouse under isoflurane anesthesia, the pumps inserted, and the incision sutured followed by 3 days of Carprofen (ER)/buprenorphine administration for analgesia. To ensure that the pump did not completely empty prior to conclusion of the studies, drug (or vehicle) was administered for 12 days in total, after which animals were euthanized.

Tissues isolated for analysis included intact eye, liver, prefrontal cortex (PFC), visual cortex (VC) and the remainder of the intact brain. For isolation of PFC and VC, regions were identified and dissected according to the method of Spijker,^16^ employing the stereotactic coordinates outlined by Paxinos and Franklin.^17^ Blood was collected by cardiac puncture using a heparinized syringe, and plasma obtained following low‐speed centrifugation. Following sacrifice, tissues were rapidly removed onto an ice‐cold glass plate, washed with ice‐cold PBS, weighed, and flash‐frozen in liquid nitrogen. Tissues were stored at −80^°^C until analysis. For mass spectrometric analysis, tissues were homogenized on ice in 0.1 mol/L perchloric acid, followed by sonication and centrifugation to remove cellular debris.

### 2.3 VGB and endogenous metabolite analysis by LC/MS‐MS

Novel methodology was developed for isolation and quantification of VGB enantiomers. For quantification, ^2^H_4_‐gabapentin (Neurontin; CAS Number:1185039‐20‐6; MW, 175.26; C_9_H_13_D_4_NO_2_) was obtained from Santa Cruz Biotechnology (Dallas, TX). S‐NIFE (*N‐(4‐Nitrophenoxycarbonyl)‐L‐phenylalanine 2‐methoxyethyl ester)* was employed for derivatization to achieve enantiomer separation (Santa Cruz Biotechnology; CAS Number:328406‐65‐1; MW 388.37; C_19_H_20_N_2_O_7_). The quantity of *S‐* and *R‐* VGB enantomers was determined in perchloric acid extracts of liver, eye, brain, PFC, and VC. Plasma samples were evaluated without perchloric acid extraction. For sample preparation, aliquots of 5‐10 μL were pipetted into glass vials and 0.01 nmol (VC, PFC and brain), 0.05 nmol (liver and plasma), or 0.2 nmol (eye) ^2^H_4_‐gabapentin added as internal standard. S‐NIFE derivatives were prepared via addition of 25 μL 62.5 mmol/L borate buffer pH 10 and 25 μL S‐NIFE (1 mg/mL in acetonitrile). Calibrators of 0, 0.001, 0.002, 0.005, 0.01, and 0.02 nmol (VC, PFC and brain), 0, 0.005, 0.01, 0.02, 0.05, and 0.2 nmol (liver and plasma), and 0, 0.02, 0.05, 0.10, 0.20, and 0.50 nmol (eye) were included in each sample batch analysis. Detected peak‐area ratios for the signals of *S* and *R* VGB were normalized to those of the internal standard and used to quantify individual VGB isomers.

Liquid chromatography (Waters Acquity) was performed using an Acquity UPLC BEH C18 1.7 μm column 2.1 × 100 mm (Waters Chromatography). A binary linear gradient from 0 to 6 minutes was used for separation of enantiomers. Solvent A consisted of 10 mmol/L ammonium bicarbonate pH 9.5 and solvent B consisted of 100% acetonitrile. The flow rate was 0.25 mL/min and the analytical column was maintained at 40°C. Detection was achieved using an AB Sciex 4000 QTrap tandem mass spectrometer equipped with an electron ion spray source (Turbo Ion Spray) operating in the positive mode. The ion source parameters were: ion spray voltage 5500 V; source temperature 400°C; curtain gas and cad gas setting 10 and 5 (arbitrary units) respectively. Multiple reaction monitoring (MRM) transitions (Q1/Q3), declustering potential (DP), and collision energy (CE) parameters were: *S* and *R* VGB: 379.1/224.1 (DP = 40V, CE = 20V) and ^2^H_4_‐gabapentin: 425.3/224.1 (DP = 45V, CE = 25V).

Total GABA and total β‐alanine were quantified in tissue extracts using solvent extraction and electron‐capture negative‐ion mass fragmentography.^18^ For these analyses, samples were acidified with 6 mol/L HCl and hydrolyzed at 110°C for 4 hours. This process serves to liberate GABA and β‐alanine from their respective storage forms, homocarnosine and carnosine (histidine dipeptides). Following hydrolysis, the samples were neutralized by adding 200 μL of 12 mol/L NaOH followed by derivatization as described.^18^ For quantitation, the molecular ions −146.1 m/z (β‐alanine‐^2^H_0_) and −149.1 m/z (^13^C_3_‐β‐alanine) were used. Creatine and 4‐guanidinobutyrate (4‐GBA) were quantified as the hexafluoroacetylacetone/PFB‐derivatives as described.^19^ Limiting plasma volumes enabled only measurement of VGB enantiomers in this matrix.

### 2.4 Statistical and data analysis

Data analysis was performed using GraphPad Prism (version 8.0; San Diego, CA). Statistical analyses employed either two‐tailed *t* test or one‐way ANOVA with post hoc analysis. Pearson correlations and linear regression analyses were performed in the GraphPad program. Determination of VGB pool size was achieved by multiplying tissue VGB concentration (*S* and *R*) by tissue weight. Tissue partition coefficients (K_p_) were calculated as the ratios of tissue to plasma VGB concentrations.^20^ For calculation of tissue VGB enantiomers as a function of dose, the actual weights of tissue (eye, brain, PFC, and VC) were used. For liver, since only a single lobe was obtained at sacrifice, published values for liver weight of 1.34 g 21 for animals of this age and gender were utilized.

## 3 RESULTS

### 3.1 Tissue and plasma distribution of racemic VGB as a function of VGB dose

**Figure 2:**
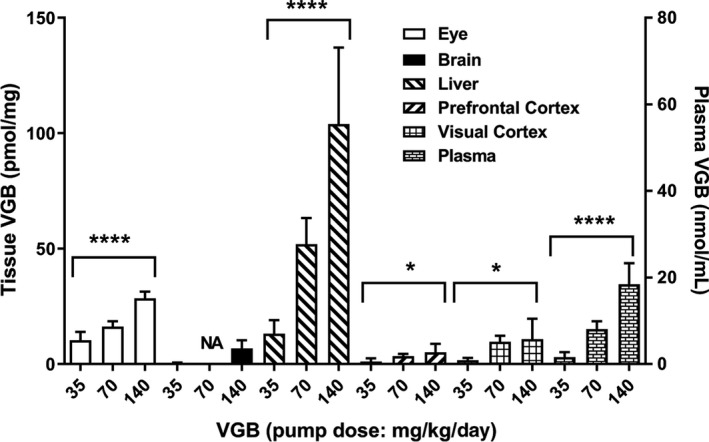
Tissue and plasma concentration of VGB as a function of VGB dose. Data for the 70 mg/kg/d dose in brain was not available. Data depicted as mean ± SEM (n = 6‐8 animals for each drug dose). Statistical analysis employed a one‐way ANOVA (*P < 0.05; ****P < 0.0001) within tissues. Tissue levels of VGB are presented as pmol/mg tissue; plasma levels in nmol/mL

The tissue and plasma concentrations of racemic VGB are shown in Figure 2 (tissue, pmol/mg wet weight; plasma, nmol/mL (μmol/L)). A significant dose‐dependent increase in concentration was observed in all tissues and in plasma: *P* < 0.0001 for eye, liver and plasma and *P* < 0.05 for PFC and VC (one‐way ANOVA). Technical error resulted in an inability to quantify brain VGB at the 70 mg/kg/d dose (NA in Figure 2). All dose‐concentration relationships were described by a linear regression model with the following slopes: eye, 0.22 ± 0.033; liver, 0.848 ± 0.09; PFC, 0.036 ± 0.01; VC, 0.078 ± 0.05; and plasma 0.298 ± 0.017 (for units see Figure 2).

### 3.2 Effect of VGB dose on endogenous metabolite tissue distribution

**Figure 3:**
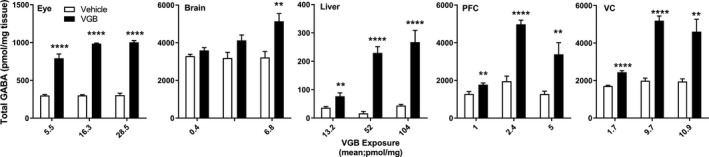
Tissue total GABA as a function of VGB dose. Data depicted as mean ± SEM (n = 6‐8 animals for each dose, vehicle and drug). Statistical analysis employed a two‐tailed *t* test (VGB vs vehicle); *P < 0.05; **P < 0.01; ***P < 0.001; ****P < 0.0001. PFC, prefrontal cortex; VC, visual cortex

**Figure 4:**
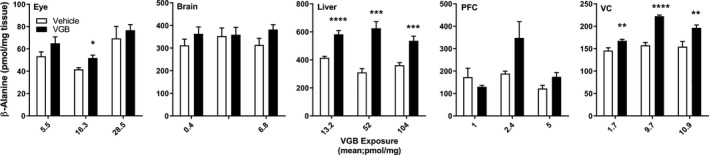
Tissue total β‐alanine as a function of VGB dose. Data depicted as mean ± SEM (n = 6‐8 animals for each dose, vehicle and drug). Statistical analysis employed a two‐tailed *t* test (VGB vs vehicle); *P < 0.05; ** *P* < 0.01; *** *P* < 0.001; ****P < 0.0001. PFC, prefrontal cortex; VC, visual cortex

**Figure 5:**
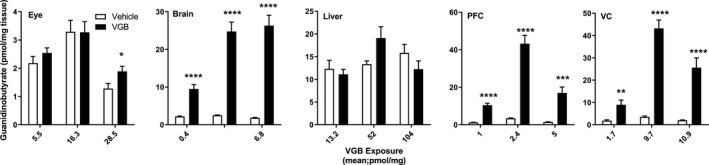
Tissue 4‐GBA as a function of VGB dose. Data depicted as mean ± SEM (n = 6‐8 animals for each dose, vehicle and drug). Statistical analysis employed a two‐tailed *t* test (VGB vs vehicle); *P < 0.05; **P < 0.01; ***P < 0.001; ****P < 0.0001. PFC, prefrontal cortex; VC, visual cortex

**Figure 6:**
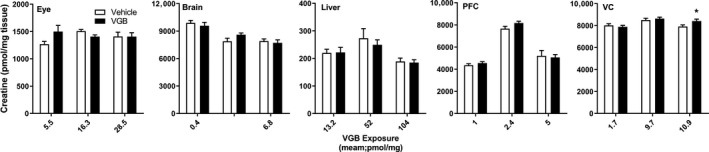
Tissue creatine as a function of VGB dose. Data depicted as mean ± SEM (n = 6‐8 animals for each dose, vehicle and drug). Statistical analysis employed a two‐tailed *t* test (VGB vs vehicle); *P < 0.05; **P < 0.01; ***P < 0.001; ****P < 0.0001. PFC, prefrontal cortex; VC, visual cortex

We examined the effect of VGB dose level on the tissue distribution of key endogenous metabolites, including total GABA, total β‐alanine, 4‐guanidinobutyrate (4‐GBA), and creatine, and these are shown in Figures 3, 4, 5, 6. GABA concentrations dose‐dependently increased in brain and liver with maximum tissue concentration observed at 140 mg/kg/d. The VGB dose‐GABA concentration curve was perfectly linear (*R* ^2^ = 1) in brain. VGB concentrations reached a maximum at 70 mg/kg/d and plateaued at 140 mg/kg/d whereas in PFC and VC, it reached a maximum at 70 mg/kg/d and decreased significantly at 140 mg/kg/d (Figure 3).

Consistent elevations with VGB exposure were observed for β‐alanine only in liver and VC (Figure 4), again with evidence of maximum effects reached at 70 mg/kg/d VGB as observed for GABA in VC. As shown in Figure 5, 4‐GBA appeared prominently only in CNS tissues (brain, PFC, VC), and only showed a significant increase in eye at the highest dose of VGB. For creatine (Figure 6), there were essentially no significant differences across all concentrations administered, and in all tissues.

### 3.3 Tissue distribution of S and R VGB

**Figure 7:**
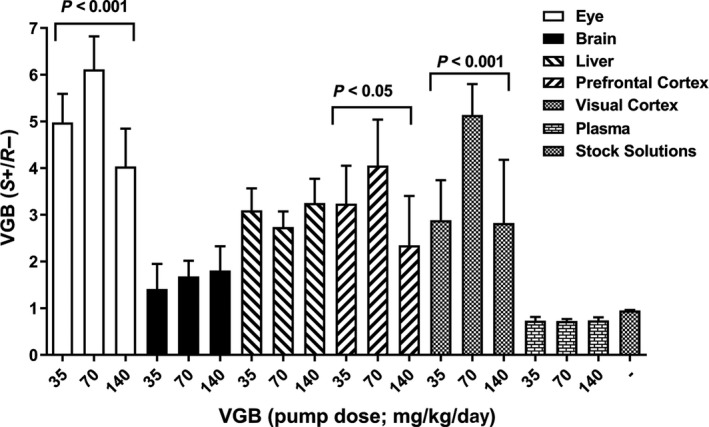
Tissue *S/R* VGB enantiomer ratios as a function of VGB dose. Data depicted as mean + SEM (n = 6‐8 animals for each drug dose). Statistical analysis employed a one‐way ANOVA within tissues

Figure 7 depicts the ratios of VGB enantiomers (*S/R*) as a function of VGB dose. This value was 0.95 (n = 2, individual values 0.94, 0.96) in stock VGB dose solutions, and 0.74 ± 0.02 (n = 14) in plasma. Strikingly higher ratios were observed at 70 mg/kg/d VGB in the eye (6.1 ± 0.29), VC (5.1 ± 0.27) and PFC (4.1 ± 0.44) (all *P* < 0.0001 compared to plasma ratios). Those ratios decreased at 140 mg/kg/d but remained much higher than plasma ratios in all three tissues: eye, 4.04 ± 0.29; VC, 2.82 ± 0.51; PFC, 2.35 ± 0.37 (all *P* < 0.0001 compared to plasma ratios). Liver enantiomer ratios were also increased above plasma ratios (approximately THREE on average) with no obvious dose‐concentration relationship (Figure 7). Total brain enantiomer ratios were only moderately increased above plasma ratios but showed a positive linear correlation with VGB dose: 1.42 ± 0.19 at 35 mg/kg/d; 1.68 ± 0.14 at 70 mg/kg/d, and 1.81 ± 0.18 at 140 mg/kg/d, suggesting a preferential enrichment of the *S* enantiomer in brain regions primarily associated with visual function.

### 3.4 Correlation of endogenous metabolite concentrations with S and R VGB enantiomers

**Figure 8:**
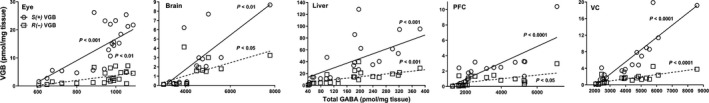
Correlations between enantiomer concentrations and the concentrations of total GABA. For correlation analyses, the data of both Figures 2 and 7 were employed throughout. For total GABA correlation, the data of Figure 3 was employed

**Figure 9:**
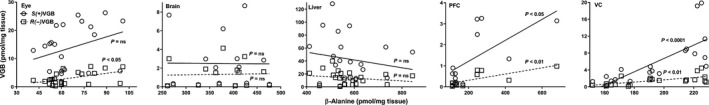
Correlations between enantiomer concentrations and the concentrations of total β‐alanine. For correlation analyses, the data of both Figures 2 and 7 were employed throughout. For total β‐alanine correlation, the data of Figure 4 was employed

**Figure 10:**
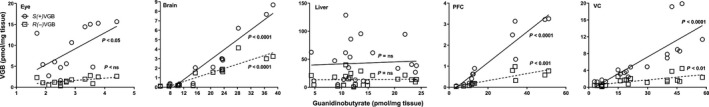
Correlations between enantiomer concentrations and the concentrations of 4‐GBA. For correlation analyses, the data of both Figures 2 and 7 were employed throughout. For 4‐GBA correlation, the data of Figure 5 was employed

**Figure 11:**
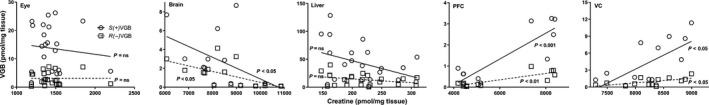
Correlations between enantiomer concentrations and the concentrations of creatine. For correlation analyses, the data of both Figures 2 and 7 were employed throughout. For creatine correlation, the data of Figure 6 was employed

The relationship between metabolites (GABA, β‐alanine, 4‐GBA, creatine) and VGB enantiomers in tissues is depicted in Figures 8, 9, 10, 11. Linear correlation between both isomers with GABA was seen in all tissues (Figure 8). For β‐alanine, linear correlations were only observed in PFC and VC (Figure 9), although there was a correlation with the *R* isomer in eye (but not for the *S* isomer). For 4‐GBA, the relationship between isomer and metabolite appeared particularly targeted to CNS tissues (total brain, PFC, and VC) (Figure 10). There was no linear correlation for either enantiomer with 4‐GBA in liver, but a significant correlation for eye between 4‐GBA and the *S* isomer (and not the *R* isomer). For creatine, there was a significant negative correlation for both isomers in brain tissue (Figure 10), consistent with the trend of decreasing creatine with increasing VGB (Figure 6). Creatine displayed a significant linear correlation with both enantiomers in the PFC and VC despite minimal effects of VGB on creatine in both tissues vs vehicle (Figure 6).

### 3.5 Tissue VGB enantiomer pools as a function of dose

**Figure 12:**
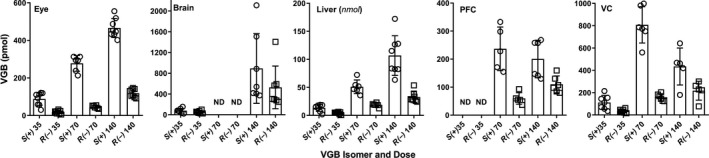
Tissue pools of VGB enantiomers as a function of VGB dose. ND, Not Determined ‐ Prefrontal cortex samples from animals treated with the 35 mg/kg/d dose were used for RNA isolation and thus were unavailable for enantiomer analysis. Data depicted as mean ± SEM (n = 6‐8 animals for each drug dose and isomer). For liver, only a single lobe was isolated at sacrifice, and thus published weights for liver of male animals of this age were employed to estimate pool size (1.34 g, based upon the data of^21^ and comparable liver weight values derived from the JAX mice website (https://www.jax.org/jax-mice-and-services). For eye, liver (note: *nmol*), and visual cortex, one‐way ANOVA for both enantiomers revealed *P* < 0.0001

These are shown in Figure 12. VGB data were not available for brain (70 mg/kg/d), and for PFC at 35 mg/kg/d. As expected, liver was the major reservoir of VGB unlike brain, PFC or VC, yet there is no reported hepatotoxicity for VGB and suggesting a specific sensitivity of the neurons of the visual system to VGB. The differences in dose‐pool size correlations for the *S* and *R* isomers in PFC, VC, and less obviously in eye were of interest. In PFC and VC, the S pool decreases at the 140 mg/kg/d dose in contrast with the linear increase in the *R* pool size with dose, resulting in a decreased *S/R* ratio as depicted in Figure 7. For eye, the pool size increase in the *S* isomer as a function of dose is not as marked as the increase in *R* pool size, translating into a decrease in the ratio as shown in Figure 7.

### 3.6 Partition coefficients (K_p_) as a function of dose and isomer

K_p_ values for *S* and *R* isomers are displayed in Table 1. For the *S* isomer, increasing VGB dose resulted in lower K_p_ values in all tissues except for VC at 70 mg/kg/d dose. A similar, but less pronounced effect, was observed for the *R* isomer. As well, the percent comparison between K_p_eye/K_p_liver revealed a dose‐dependent effect for both isomers (35 mg/kg/d, 46 and 28% (*S:R*); 70 mg/kg/d, 37 and 16%; and 140 mg/kg/d, 31 and 18%).

**Table 1:** Partition coefficients (tissue/plasma) as a function of VGB enantiomer (*S/R*)^a^

Tissue	35 mg/kg/d	70 mg/kg/d	140 mg/kg/d
Eye	3.60; 0.53	2.17; 0.26	1.55; 0.28
Brain	0.18; 0.09	NA	0.30; 0.12
Liver	7.84; 1.87	5.90; 1.60	4.96; 1.56
PFC	0.62; 0.14	0.42; 0.08	0.24; 0.08
VC	0.97; 0.25	1.26; 0.18	0.55; 0.14

a Vigabatrin content was not available (NA) for brain at the 70 mg/kg/d dose. Calculations were based upon a mean plasma ratio (*S/R*) of 0.74. Under each dose (column), the numerical values separated represent the *S‐(+)* and *R‐(‐)* isomer partition coefficients respectively. For each value, n = 6‐8 animals were employed.

## 4 DISCUSSION

VGB is currently used as adjuvant therapy for refractory epilepsies, complex partial seizures, secondarily generalized seizures, infantile spasms, and is under active investigation in tuberous sclerosis complex.^2^, ^22^, ^23^, ^24^ However, an extensive body of literature suggests that VGB is associated with peripheral visual field defects (pVFD),^25^ but others have suggested that VGB ocular toxicity correlates with pre‐existing anomalies, both structural and genetic.^26^ The occurrence of permanent visual field constriction in patients receiving VGB is ~6‐7%).^23^, ^27^, ^28^ This estimation may be an underestimate, since measures of VGB‐associated ocular toxicity relies on insensitive serial funduscopic exams or electroretinograms (ERG), or investigational ocular coherence tomography. There is no consensus as to what testing is sensitive enough to detect early onset pVFDs and even ERGs are debatable, since the normal findings change with development, and robustness of findings in infants remains questionable.

Long‐term VGB intervention associates with peripheral atrophy of the retinal nerve fiber layer,^29^, ^30^, ^31^, ^32^ and rodents similarly treated manifest disorganization of the photoreceptor nuclear layer and cone photoreceptor damage.^25^, ^33^ It has been suggested that VGB‐induced elevation of ocular/retinal GABA induces excitotoxicity via GABAergic receptors,^34^, ^35^, ^36^ resulting in oxidative stress.^5^, ^10^, ^15^, ^44^ Other studies have suggested globus pallidi and white matter anomalies associated with chronic VGB intake,^41^, ^42^, ^43^ and pathological roles for amino acids (ornithine, taurine) that share structural and biochemical properties of GABA have also been implicated in VGB‐associated ocular toxicity.^29^, ^44^, ^45^ Thus, there is no clear consensus as to the mechanism(s) of potential VGB ocular toxicity.

In addition to evaluating dose‐dependency of VGB at steady‐state, a further innovation of our study included evaluation of metabolites associated with VGB intervention, including GABA. These included β‐alanine and 4‐GBA, the former a substrate for GABA‐T activity,^7^ and the latter a metabolite known to accumulate with VGB intervention^46^ and in SSADHD,^6^, ^8^ the latter featuring GABA accumulation. To ensure accurate quantitation of these intermediates, we measured total GABA and total β‐alanine, an important consideration since both intermediates can be stored as the L‐histidine dipeptides, homocarnosine, and carnosine respectively.

The significant increase in total GABA only at 140 mg/kg/d likely reflects a low brain penetration of the drug, confirmed by our estimate of brain VGB pool size. For 4‐GBA, prominent VGB dose‐dependent increases appeared restricted to the CNS, although a significant increase was observed in eye at 140 mg/kg/d. The latter is consistent with other studies which demonstrated VGB accumulation in rat retina, although the isomeric distribution was not determined.^47^ With the exception of the high‐dose of VGB in VC, there was no effect of any dose of VGB on creatine in any tissue, arguing against the proposal that GABA interferes with the AGAT reaction. Nevertheless, there were significant linear correlations between *S* and *R* isomers and VGB for brain creatine (negative correlation), in addition to strong positive linear correlations in PFC/VC for creatine. As an organic cation, creatine would partition from plasma to brain on organic cationic transporters at the blood‐brain barrier, and high level VGB might block this process, either from plasma or CSF, although this is an untested hypothesis.^48^ Another potential source of 4‐GBA resides in the metabolism of the diamine agmatine,^49^ an intermediate involved in urea cycle function and the metabolism of diamines that has not been implicated in VGB ocular toxicity.

The role(s) of GABA, β‐alanine and 4‐GBA in the ocular toxicity of VGB remain unknown. Conversely, we and others have demonstrated that supraphysiological GABA impacts the mTOR pathway of autophagy, leading to mitochondrial accumulation and enhanced oxidative stress.^10^, ^11^, ^50^, ^51^, ^52^ β‐Alanine, the structural homologue of GABA, has GABAergic and glycinergic roles that are well‐described,^53^ but its role in VGB‐mediated toxicity has not been investigated. 4‐GBA can also give rise to an internal lactam structure, the neurochemical properties of which remain to be explored. It is also tempting to speculate that β‐alanine and taurine, the latter implicated in VGB‐mediated ocular toxicity, have structural similarities in which taurine has replaced the carboxylic acid group of β‐alanine with a sulfonic acid residue.

Schousboe et al^15^ documented stereoselective uptake of VGB isomers (preferentially the *S* isomer) in cultured neurons and astrocytes. Here, we observed the highest accumulation of the *S* isomer in eye and VC, closely followed by PFC. To extend those studies, we correlated metabolites with VGB isomer. Total GABA significantly correlated with both isomers in all tissues. For β‐alanine, correlations of both isomers was observed in PFC and VC, although we observed a mildly significant correlation with the *R* isomer in eye. For 4‐GBA, there were significant correlations for both isomers in brain, PFC and VC, and interestingly a significant correlation in eye for only the *S* isomer. This may suggest a more prominent role for 4‐GBA and the active isomer of VGB in ocular toxicity, and it will be important to examine these roles in isolated retina. Finally, we found that the pool of VGB in eye was not significantly different from that in brain and VC at the 35 mg/kg/d dose, and was also significantly different from the PFC VGB pool at the 70 mg/kg/d dose.

A concern with our methodology (acid extraction followed by LC‐MS/MS) was the possibility of selective extraction of VGB in different tissues during sample processing. A priori, there is no reason to assume that extraction efficiencies for *S‐(+)* and *R‐(‐)* VGB should be extensively different. Conversely, the different matrices (eye, brain, and liver) could certainly result in differential VGB extraction, based upon structural considerations. For eye and liver, the relative ratio of protein:lipid would be predicted to be higher than that of brain, potentially altering extraction efficiency. We had hoped to address this potential confound using stable‐isotope labeled VGB (in lieu of gabapentin), enabling isotopically labeled VGB to be added to tissues prior to extraction to gauge extraction efficiency. However, we found that commercial preparations of labeled vigabatrin (^13^C, ^2^H_2_) demonstrated insufficient isotope enrichment, making data calculation a challenge. On the other hand, tissue distribution of VGB (liver > eye ~ plasma > brain (including both PFC and VC)) mirrored distributions predicted for brain penetration across the blood brain barrier, suggesting that extraction efficiency across tissues was comparable.

In conclusion, this study represents the first examination of the tissue distribution of VGB isomers in a mammalian species, and the first attempt to correlate enantiomer content with metabolite content known to be influenced, or potentially influenced, by VGB. The preferential accumulation of the active *S* isomer in eye and VC, exceeding that in other brain regions, may contribute to heightened GABA‐T inhibition in tissues implicated in visual function compared to other tissues or brain regions, perhaps explaining the selective ocular toxicity of the drug. Our results also may challenge the concept that VGB does not isomerize in vivo,^1^ although this would require measure of specific enantiomers following either *R* or *S* administration. Further, it is possible that selected tissues/regions (eye, PFC, VC) preferentially transport the *S* isomer,^15^ thereby further enhancing GABA accumulation and increasing off‐target effects in eye and other tissues. Tissue transport considerations suggest that characterization of ocular transporters potentially moving VGB (eg, *SLC16A8*, confined to retinal pigment and choroid plexus epithelium) may be highly relevant as a follow‐up investigation to our study. Furthermore, it will be of interest to determine the VGB content of aqueous and vitreous humor, and retina using our method, with the prediction that the *S‐(+)* isomer will significantly concentrate in retina. Until such time as the issue of ocular toxicity of VGB is clarified, or mechanisms defined, caregivers will need to continue to cautiously monitor risk‐benefit associations with this unique antiepileptic agent.^54^

## AUTHOR CONTRIBUTIONS

*Participated in research design:* Walters, Ainslie, Schmidt, Roullet, Gibson. *Conducted experiments:* Walters, Jansen, Salomons, Brown. *Contributed new reagents or analytical tools:* Ainslie. *Performed data analysis:* Ainslie, Roullet, Gibson. *Wrote or contributed to the writing of the manuscript:* Walters, Jansen, Ainslie, Brown, Roullet, Gibson.

## DISCLOSURE

None declared.
